# Exploring health worker absenteeism at public healthcare facilities in Chhattisgarh, India

**DOI:** 10.1017/S1463423624000343

**Published:** 2024-10-17

**Authors:** Priyanka Kerketta, Karthika Maniyara, Edukondal Palle, Prakash Babu Kodali

**Affiliations:** Department of Public Health and Community Medicine, Central University of Kerala, Periye, Kerala, India

**Keywords:** health worker absenteeism, human resources for health, health systems, healthcare delivery, primary health care

## Abstract

**Aim:**

This study aims to assess the health worker absenteeism and factors associated with it in a high-focus district in Chhattisgarh, India.

**Background:**

Human resources for health are among the key foundations to build resilient healthcare systems. Chhattisgarh is a high-focus Indian state with a severe shortage of health care workers, and absenteeism further aggravates the shortage.

**Methods:**

This study was conducted as a mixed-methods study employing sequential explanatory design. Absenteeism was defined as the absence of health worker in the designated position without a formal leave or official reason in two different unannounced visits. A facility survey across all the public healthcare facilities in Jashpur district, Chhattisgarh, was conducted through random, unannounced visits employing a checklist developed based on Indian Public Health Standards. Twelve participants were purposively sampled and interviewed from healthcare facilities to explore factors associated with absenteeism. Survey data were analysed descriptively, and thematic analysis was employed to analyse qualitative interviews.

**Findings:**

Among all the positions filled at primary health centre level (*n* = 339), close to 8% (*n* = 27) were absent, whereas among the positions filled at community health centre level (*n* = 285), only 1.14% (*n* = 4) were absent. Absenteeism was not found in the district hospital. Qualitative interviews reveal that macro-level (geographical location and lack of connectivity), meso-level (lack of equipment and amenities, makeshift health facilities, doctor shortage, and poor patient turnover), and micro-level (unmet expectations) factors contribute to health worker absenteeism.

**Conclusion:**

Health worker absenteeism was more at PHC level. Systemic challenges, human resource shortages, and infrastructural shortcomings contributed to health worker absenteeism.

## Background

Human Resource for Health is an essential building block of the health systems. The availability of health workers is an important determinant for building and sustaining resilient health systems. The health systems in low- and middle-income countries (LMICs) are poised with challenges of health worker shortages and unavailability of health workers in the healthcare facilities. Indian public health system has a chronic shortage of skilled health workers (Karan *et al*., 2021). In addition, the skewed distribution of health workforce across the states and rural-urban regions implies that some of the states have a lower net-availability of health workers. While Indian Public Health Standards (IPHS) outline minimum requirement of health workers per healthcare facility, the health facilities in several states are understaffed, with sanctioned positions lying vacant, resulting in a health worker shortage.

The recent Rural Health statistics of India report an average shortfall of 6.8% for allopathic doctor availability at the primary health centre (PHC) level and 63.3% shortfall for specialists at the community health centre (CHC) level in the country (NHM, 2020). The problem of health worker shortage is further aggravated by absenteeism among health workers. The health worker absenteeism may be defined as “*a phenomenon where the health worker is being absent from his/her duties in the healthcare facility without taking a formal leave or not reporting to work for non-professional reasons*” (Chaudhury and Hammer, 2004). Health worker absenteeism compromises health service delivery, creates excessive workload for existing health workers, demotivates the healthcare staff, and minimises the efficiency of the public health system (Kisakye *et al*., 2016). Health worker absenteeism can erode the patient’s trust on public health system and instigate the patients to seek healthcare from private practitioners and under-trained healers (Hall, 1998). Studies in LMICs (Kenya, Bangladesh, Uganda, India, Peru) showed high rates of health worker absenteeism ranging between 25% and 40% (Chaudhury *et al*., 2006; Muthama *et al*., 2008).

In the Indian context, remote rural areas with limited health system capacity often face the brunt of limited health worker availability. The state of Chhattisgarh is considered as one of the Empowered Action Group (EAG) States in India with shortfall in key health and development indicators. According to the recent National Institute for Transforming India (NITI-AYOG) report on multidimensional poverty, Chhattisgarh was ranked seventh in the country with 29.91% of population multi-dimensionally poor, 43.02% of population undernourished, and 24.7% of the mothers deprived of adequate maternal health (NITI-AYOG, 2021). More than 1/3rd of the state’s total population is tribal population. The Jashpur district in Chhattisgarh is among the high-focus districts, with over 62.28% of the population being tribal population. The district ranks fifth within the multi-dimensional poverty index of the state, with over 45% of the district’s population estimated to be multi-dimensionally poor (NITI-AYOG, 2021). The health system within the state of Chhattisgarh faces similar infrastructural and operational challenges as several low-income country settings internationally. Very little research is conducted concerning health worker absenteeism in India in general and EAG states in particular. Considering the strides taken by the country to achieve sustainable development goals, understanding health worker absenteeism in the context of Chhattisgarh can contribute to strengthening the health system regionally and nationally. Moreover, given the trying nature of the state’s geography and the challenges health system faces, it can be said that findings could be of importance internationally in similar low-resource settings. Given the context, this study aims to address the following objectives:

1. To assess the health worker absenteeism and availability at public health facilities in Jashpur District, Chhattisgarh.
2. To explore the factors influencing health worker absenteeism in public health facilities in Jashpur District, Chhattisgarh.

## Methods

### Research design

We conducted a mixed-methods study adopting a sequential explanatory design (Ivankova *et al*., 2006). The quantitative phase employing a facility survey of public healthcare facilities in the district preceded the exploratory qualitative phase. The facility survey collected data on health worker positions filled and health worker absenteeism using a checklist. In-depth interviews of purposively sampled health workers enabled exploration of factors influencing health worker absenteeism.

### Study sample

The study sample comprised public healthcare facilities across Jashpur District, Chhattisgarh. In 2019, the district had a total of 43 government health centres and hospitals (excluding sub-centres and camp clinics), with 34 PHCs, eight CHCs, and one district hospital. All the 43 public health facilities were included for facility survey. The qualitative sample comprised 12 health workers who were purposively sampled from the three types of health care facilities (PHCs, CHCs, and district hospital). The participants for qualitative phase comprised chief medical and health officer from the district hospital, block medical officer from CHC, rural medical assistants (RMAs), staff nurses, lab technicians, and pharmacists representing various health worker cadres.

### Data collection tools

The data collection tools were employed for quantitative and qualitative phases of the study separately. For collecting the data on positions filled and health worker absenteeism, we developed a checklist based on Indian Public Health Standards (IPHS) for the availability of health workers in the public health facilities (i.e., at the PHC, CHC, and district hospital) (NHM, 2012). We conducted unannounced visits to ascertain health worker absenteeism. Unannounced visits are considered appropriate approach to verify attendance and directly observe the presence of health workers in their designated positions (Cheriyan *et al*., 2010; Muralidharan *et al*., 2011; Fujii, 2019). While acknowledging the inherent challenges of unannounced visits related to randomness, generalisability, privacy, and observer bias, we mitigated these issues by focussing our study on a single district. Our approach included sampling all health centres within the district, obtaining necessary approvals, and implementing an unannounced visit process using a structured checklist. The checklist was used to mark for the number of positions filled for each health worker cadre against the sanctioned posts and assess the health worker’s presence/absence against a filled position (see Supplementary File 1). The absenteeism was assessed at each cadre level and for each type of healthcare facility. A health worker was considered to be absent from his/her position if the health worker was unavailable in the healthcare facility/position without any official reason or a pre-approved leave in at least two unannounced visits within the duration of one month. The qualitative interviews were conducted using in-depth interview guides to explore the health worker’s views on absenteeism. The in-depth interview guides explored the health worker absenteeism, the reasons for absenteeism, the challenges faced due to health worker absenteeism, and measures to contain health worker absenteeism. The content and face validity of the tools were assessed prior to employing them for data collection.

### Data collection

The data collection was conducted between December 2018 and March 2019. The data were collected sequentially, wherein the collection and analysis of quantitative data preceded qualitative data collection. The quantitative data were collected through facility survey employing unannounced visits of healthcare facilities on the working days. Two unannounced visits per healthcare facility on random days were conducted within a span of 1 month. In each visit, the health worker’s absence was confirmed by cross-referencing with various documents, including the attendance register, leave records, meeting notices, posting orders, circulars, request letters, and duty roster. In cases where paperwork was unavailable, an oral confirmation from the supervising authority at the health centre was obtained to verify the absence. A health worker was considered to be absent if they were absent without any reason for two random, unannounced visits. Absenteeism was assessed only in the positions where the position for a particular cadre of health worker was filled. The checklist developed based on the IPHS standards was filled by the trained researcher, who was a Master of Public Health graduate.

The qualitative data were collected using face-to-face, in-depth interviews. The interviews were audio-recorded with a prior consent. All the interviews were conducted in the vernacular language. The interviews were conducted in the health facility or in the participant’s office. The duration of interviews ranged from 40 to 60 minutes, with an average duration of 48 minutes.

### Data analysis

The data from facility survey were analysed descriptively. The percentage of filled positions and percentage of absenteeism among filled positions across the health worker cadres and healthcare facility types were analysed descriptively.

Health worker absenteeism was computed using the formula 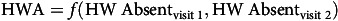 , where HWA = health worker absenteeism; HW = health worker.

The percentage of health worker absenteeism for each cadre is computed using 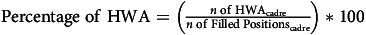 , where 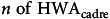 = total number of health workers absent in specific cadre, and 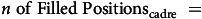 total number of filled positions for specific cadre.

The percentage availability of health worker was computed as 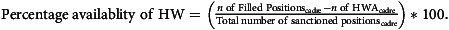


The qualitative interviews were analysed employing the thematic analysis approach (Braun and Clarke, 2006). The in-depth interviews were coded inductively. The interviews were first familiarised through the process of transcription, translation, reading, and re-reading. The primary codes were developed as descriptive codes and process codes. Further secondary codes were developed from the primary codes, and then themes were developed from the secondary codes. The triangulation was conducted at the level of data collection (identification of healthcare facilities for qualitative sampling) and in the interpretation phase of the study.

## Results

### Quantitative results

**Figure 1.**
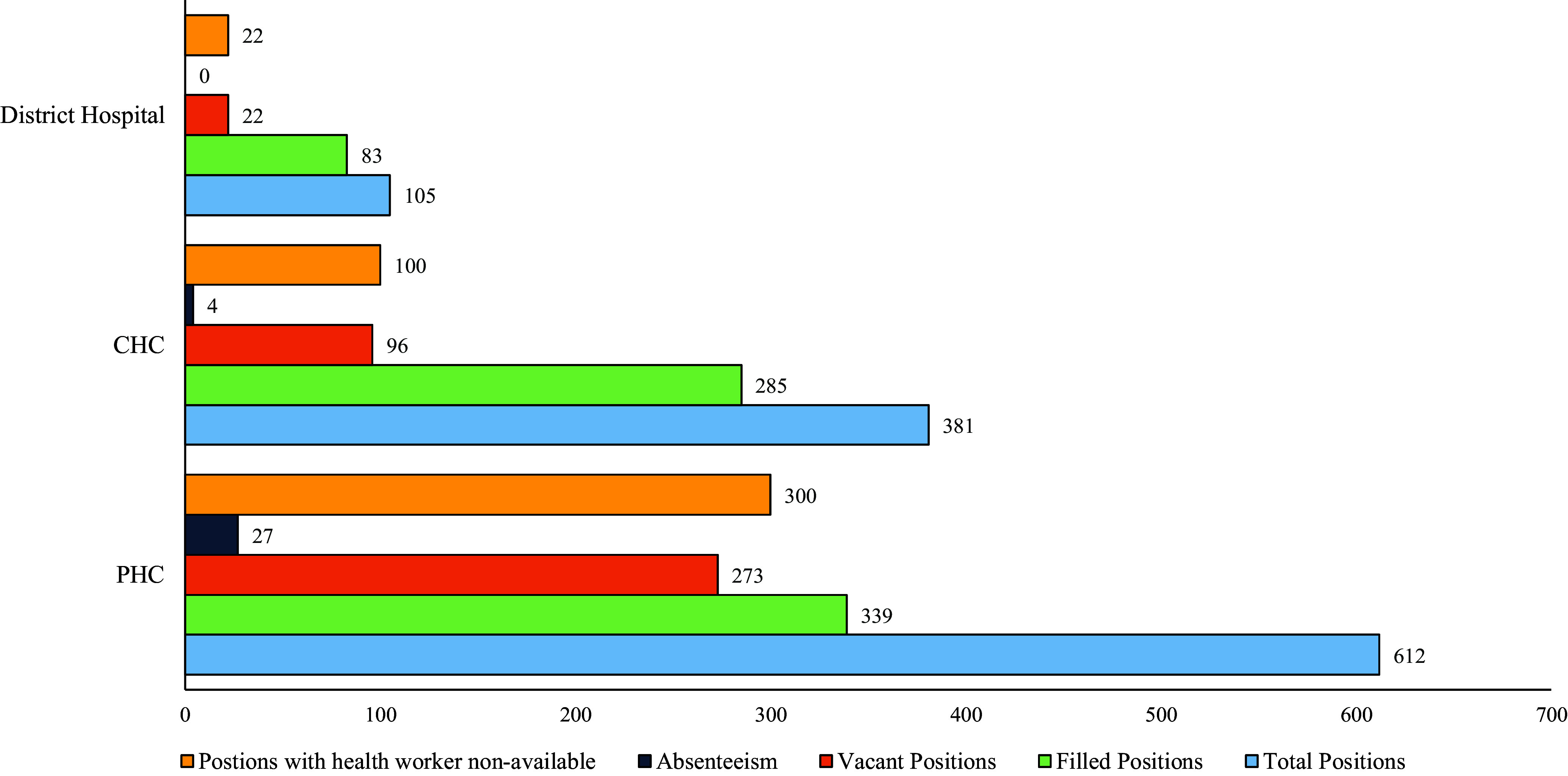
Figure outlining the health worker availability and absenteeism in health facilities. Positions with health workers non-available = (Vacant positions + Absenteeism); PHC = primary health centre, CHC = community health centre; source (primary study).

A total of 43 public health facilities were surveyed employing unannounced visits. The district hospitals did not have any absenteeism, while absenteeism across all cadres of health workers was 8% and 1.14% at PHCs and CHCs, respectively. In district hospital, close to a quarter of the positions were unfilled. In PHCs and CHCs, more than a third of positions across healthcare facilities were unfilled (see Figure 1).

### Health worker absenteeism

Among PHCs, all the existing positions were filled only for the cadre of RMAs. For the cadres such as medical officers at PHCs and Ayurveda, Yoga, and Naturopathy, Unani, Siddha, and Homeopathy (AYUSH) pharmacists, the percentage of filled positions was less than 25%. Among cadres at least half-filled, RMA, AYUSH Medical Officers, and lab technicians had more than 10% absenteeism (see Table 1). The absenteeism was observed to be less among CHCs compared to PHCs. Staff nurses (3.5%) and ward boys (3.5%) were cadre with absenteeism observed (see Table 1). The district hospital did not have any absenteeism among the health workers. However, it was observed that the positions for several cadres at CHC and district hospital level are either partially filled or unfilled (see Supplementary File 1).

**Table 1.** Health worker absenteeism by cadre at PHCs and CHC

	Health worker cadre	Positions	Filled	1st Follow-up	2nd Follow-up	Number absent	% Absenteeism	% Availability
PHC (*n* = 34)	Medical officer	34	8	1	0	0	0.0	23.5
	AYUSH medical officer	34	26	5	4	4	15.4	64.7
	Rural medical assistant	34	34	4	4	4	11.8	88.2
	Data entry operator	34	13	2	1	1	7.7	35.3
	Pharmacist	34	31	2	2	2	6.5	85.3
	AYUSH pharmacist	34	7	3	2	2	28.6	14.7
	Staff nurse	102	86	7	4	4	4.7	80.4
	Health worker female	34	30	3	3	3	10.0	79.4
	Health worker male	34	21	3	1	1	4.8	58.8
	Lady health visitor	34	15	0	1	0	0.0	44.1
	Lab technician	34	26	3	5	3	11.5	67.6
	Group D worker	68	19	3	2	2	10.5	25.0
	Sanitary worker	34	23	1	1	1	4.3	64.7
	Total		339			27	8%	
CHC (*n* = 8)	Block medical officer	8	8	0	0	0	0.0	100.0
	General surgeon	8	5	0	0	0	0.0	62.5
	Physician	8	8	0	0	0	0.0	100.0
	Obstetrician and gynaecology	8	8	0	0	0	0.0	100.0
	Paediatrician	8	2	0	0	0	0.0	25.0
	Dental surgeon	8	4	0	0	0	0.0	50.0
	General duty medical officer	16	16	0	0	0	0.0	100.0
	Medical officer AYUSH	8	8	0	0	0	0.0	100.0
	Staff nurse	90	85	4	5	3	3.5	91.1
	Pharmacist	8	8	0	0	0	0.0	100.0
	AYUSH pharmacist	8	8	0	0	0	0.0	100.0
	Lab technician	18	18	0	1	0	0.0	100.0
	Radiographer	8	7	0	0	0	0.0	87.5
	Cold chain and vaccine logistic assistant	8	8	0	2	0	0.0	100.0
	OT technician	8	7	0	0	0	0.0	87.5
	Registration clerk	16	14	0	0	0	0.0	87.5
	Statistic Assistant/data entry operator	16	16	0	0	0	0.0	100.0
	Account assistant	8	8	0	0	0	0.0	100.0
	Dresser	8	8	0	0	0	0.0	100.0
	Ward boy	40	28	3	1	1	3.6	67.5
	Driver	11	11	1	2	0	0.0	100.0
	Total		285			4	1.14%	

*Note*: The table provides details of absenteeism among the health worker cadres, which are at least partially filled at the at the PHC and CHC.

AYUSH = Ayurveda Yoga Unani Siddha and Homeopathy; CHC = community health centre; OT = operation theatre; PHC = primary health centre.

### Qualitative results

**Figure 2.**
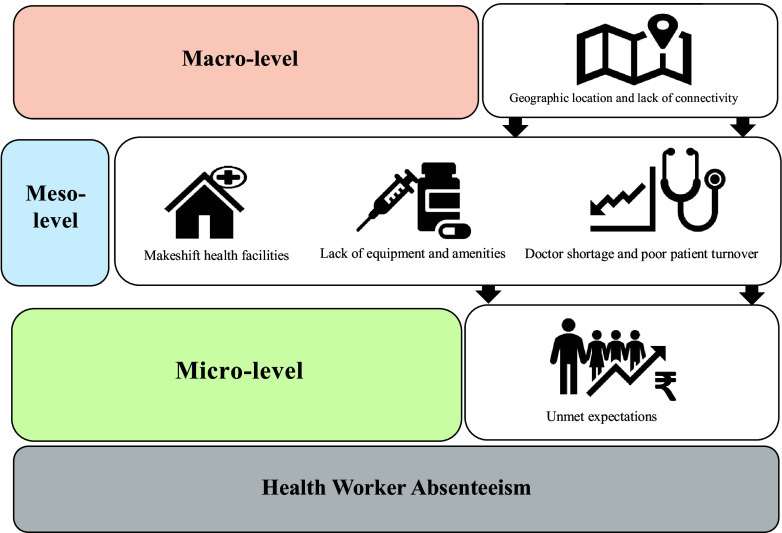
Factors influencing health worker absenteeism: findings of qualitative phase. Source (primary study).

The in-depth interviews were conducted to explore the factors influencing absenteeism among health workers. The themes were developed reflecting various macro-, meso-, and micro-level factors influencing health worker absenteeism. At a macro level, a) geographical location and lack of connectivity; at a meso level, b) lack of equipment and amenities, c) makeshift healthcare facilities, d) doctor shortage and poor patient turnover; and at a micro-level, e) unmet expectations impacted health worker absenteeism. Each of the identified factors by themselves and reinforcing others result in health worker absenteeism (see Figure 2).

#### Theme 1: geographical location and lack of connectivity

At a macro level, health worker absenteeism was attributed to geographical location of healthcare facility and road network. The respondents reported that forest terrain, lack of public transport, poor roads, and connectivity issues were reasons for absenteeism. One health worker at a PHC reported that:

*This area is largely covered with dense forests and wild animals. So, we cannot travel alone its very risky. There is no proper transport facility as well* (Respondent 04)

Connectivity issues were further amplified during specific seasons such as monsoon, wherein overflowing rivers limit connectivity. One participant reported the following.

*Transportation problem is the main reason of being absent, road is not good. Especially in rainy season we cannot travel, water is flowing over the bridge, and we have only one way to come to the hospital.* (Respondent 03)

#### Theme 2: lack of equipment and amenities

Within the healthcare facilities, the lack of equipment and absence of basic amenities fuelled demotivation and contributed to health worker absenteeism. It was reported that hospital equipment was either unavailable or damaged, compromising the health worker’s ability to perform their roles effectively.

*In rural and very interior area we don’t get interest to do our job because of lack of basic facilities. Hospital equipment are damaged and we are not getting new ones, these all things have to be replaced so that we can work properly*. (Respondent 04)

Moreover, it was observed that some health facilities lacked most basic amenities such as electricity, running water, functional toilets, and so on, resulting in high absenteeism among the health workers.

*Without any proper management it is as if we are giving an infection to the patient. Because there is no electricity here, we cannot even boil the instrument*. (Respondent 06)

#### Theme 3: makeshift health facilities

Several PHCs were functioning from non-PHC setups, shared premises with other government offices, and panchayat bhavans. Working in these makeshift facilities was also found to be a reason for absenteeism. One health worker reported

*Infrastructure problem is contributing to absenteeism here; we don’t feel to work without a hospital building. It’s very difficult to make it as hospital because this is panchayat bhavan not a hospital building*. (Respondent 03)

Additionally, makeshift health facilities are often situated in areas that are challenging to access and typically lack essential supportive infrastructure, connectivity, and accommodations. This situation contributes substantially to staff absenteeism. A health worker described the predicament as follows:

*Health workers can’t travel from the home to hospital because very less buses are available and there are no available quarters in the facility to stay*. (Respondent 08)

#### Theme 4: doctor shortage and poor patient turnover

In facilities experiencing significant absenteeism among health workers, a critical factor identified is the low rate of patient visits. Poor patient turnover rate was found to be a demotivator for health workers, contributing to their absenteeism.

*Because patients are not coming to the hospital due to lack of facilities and health workers, the health workers are also not coming for the duty because it’s like simply coming and sitting in the hospital*. (Respondent 05)

In support of the arguments, one of the major factors preventing patients from utilising health facilities was a shortage of medical doctors and adequate equipment. One RMA reported as follows:

*MBBS doctor should be in our facility, only then people will come for the treatment. People do not trust RMA. Patients are not coming to the hospital after seeing lack of facilities here, people need qualified MBBS doctors to prescribe medicine*. (Respondent 02)

#### Theme 5: unmet expectations

At an individual level, the unmet personal and professional expectations contributed to high health worker absenteeism. Inadequacy of health workers contributing to higher workloads as a plausible reason for absenteeism was reported by several participants.

*We don’t have enough man power within our facility. You can see the positions vacant. Government has to improve the facilities of all the public hospitals with adequate human resource*. (Respondent 01)

In addition to workload due to health worker shortages, unmet financial and professional expectations function as a contributor to health worker absenteeism.

*Government should increase salary so that we will get motivated to work, they should provide proper road, good water facility, separate building for staff*. (Respondent 11)

The data gathered from interviews reveal that health worker absenteeism is a multifaceted issue influenced by multiple factors that span the individual, systemic, and geographical spectrums. On a broader scale, certain geographical challenges in regions of Chhattisgarh present formidable obstacles in establishing sufficient healthcare infrastructure and ensuring connectivity. At the intermediate, or healthcare system level, there are notable deficiencies in human resources, specifically medical doctors, as well as in the availability of infrastructure, equipment, and the delivery of health services. These systemic inadequacies not only deter patients from seeking medical care but also collectively demoralise health workers, further contributing to absenteeism.

## Discussion

Our study found that the average percentage of absenteeism across cadres among PHC staff was 9% and 0.33% among CHC staff. Our study’s finding is lower than earlier estimates of over 30% of health worker absenteeism documented in India (Chaudhury *et al*., 2006; World Bank and International Monetary Fund, 2008; Muralidharan *et al*., 2011). The difference in our study could be attributable to progress made due to substantial investments into health systems strengthening under National Rural Health Mission (NRHM) and National Health Mission (NHM) and our emphasis on a single high-focus district. Nevertheless, close to a third of health workers not being available either due to unfilled positions or absenteeism reflects a poor resilience of the public healthcare system. While the overall absenteeism is minimal, health worker availability is seriously compromised by unfilled/vacant positions. Evidence indicates that non-availability of healthcare workers reduces the odds of public health facility use, increases the odds of using private care facilities, and procures medicines from unlicensed providers (Zhang *et al*., 2021).

Our study found that the health worker absenteeism is a problem at the PHC level compared to CHC or district hospital. This finding is of prominence given that PHCs play an important role in health promotion, disease prevention, and delivery of essential healthcare services at grassroots. This finding is in contrast to the published literature in other LMIC context, which reported that health workers in district-level healthcare facilities have higher absenteeism (Muthama *et al*., 2008). The observed difference in absenteeism between PHCs and district hospital can be primarily attributed to the geographical location of the healthcare facility, followed by better human resource management practices at the district hospital level. PHCs such as “Kolhenjhariya” had over 40% of their health workers absent during the unannounced visits. Incidentally, the said PHC is 56 km (approximately 34.8 miles) from the district headquarters, requiring travel in challenging terrain. Two out of the top three PHCs in terms of absenteeism had a distance from district headquarters of >80 km. An earlier study from Nigeria on absenteeism among hospital workers reported that travel and transportation issues accounted for 4.4% of absenteeism among health workers (Isah *et al*., 2008). Moreover, from the observations made in qualitative interviews, it could be argued that geographical accessibility of healthcare facilities contributes substantially to health worker absenteeism. The district hospital, which was located within the district headquarters, did not face similar operational challenges, which might be a reason for low absenteeism at district level.

A key observation made was a close link between the health worker shortage (in terms of the number of vacant positions) and health worker absenteeism. Evidence from India points that unfilled positions are an occupational stressor for in-position health workers (Tripathi *et al*., 2010). Vacant positions could potentially contribute to health worker absenteeism through occupational burnout. A prominent finding made was concerning the shortage of medical doctors at the PHC level. Less than a quarter of the positions of medical doctors at the PHC level were filled. Qualitative interviews indicate that shortages of doctors at PHCs contribute to low patient turnover despite the availability of other health workers, such as RMAs. Several LMICs have experimented with mid-level care providers as a potential replacement for non-availability of medical doctors in rural remote locations (Moola *et al*., 2020). In Chhattisgarh, the health worker cadre recruited as such were RMAs (Sundararaman *et al*., 2008). While mixed evidence exists on the patient acceptance and effectiveness of mid-level health care providers (MLHP), the amount of patient’s trust that could be generated by an RMA compared to a professional medical doctor is contended. Evidence suggests that patients’ trust on health workers influences the health service utilisation (Mallari *et al*., 2020; Netemeyer *et al*., 2020). We found in our qualitative phase that shortage of medical doctors at PHC result in lower utilisation, in turn resulting in inattentiveness and absenteeism among the existing health workers. A recent study estimated that increasing the availability of medical doctors in government healthcare facilities would increase the utilisation of outpatient services for fever cases by up to 50% (Iles, 2019). Our study observations also strengthen the argument towards the irreplaceable nature of a medical doctor in building public perception towards government healthcare facilities. While available published literature does not report on influence of medical doctor availability on absenteeism on health workers, from our study’s findings, it can be argued that medical doctor shortage can contribute to health worker absenteeism by influencing the patient turnover rate and health worker burnout.

Another key finding of the study is the lack of infrastructure at health facility as a contributor to health worker absenteeism. The study observed that health workers were demotivated to work from the makeshift healthcare facilities in panchayat bhavans and village offices. The delay in provision of an adequate working space, coupled with associated challenges such as lack of equipment, absence of basic amenities, and non-availability of the residential facilities for the healthcare staff, were reported to be resulting in health worker absenteeism. Research from other developing countries reported the prominence of unsatisfactory work conditions in increasing the odds of health worker absenteeism (Mudaly and Nkosi, 2015). According to recent estimates, more than 14.5% of the PHCs in Chhattisgarh function through makeshift methods (NHM, 2020). Moreover, a significant proportion of these makeshift healthcare facilities were from remote rural and tribal regions facing the challenges of accessibility and health worker shortage. While the makeshift health facilities might address the issue of availability of healthcare services, these solutions are not sustainable alternatives as they can hinder the efficient functioning of the health system, as witnessed in this study.

A multi-pronged approach is needed to prevent health worker absenteeism and improve health worker availability in the public healthcare system. Firstly, the health system has to be strengthened by providing the necessary financial and infrastructural inputs. Strengthening the health system will improve the patient turnout and working conditions of the health workers. Secondly, the recruitment process should focus more on filling the gap in terms of short-fall positions; this will improve the availability of health workers. Thirdly, the health system should insist on utilising measures to monitor health worker absenteeism (usage of electronic/biometric attendance monitoring systems), provide supportive services (such as residential quarters or transportation facilities), and initiate measures to encourage health worker attendance (through incentives) in difficult to reach geographies.

### Limitations of the study

The survey portion of the study was cross-sectional, which prevents us from capturing the incidence of absenteeism across the healthcare facilities. It was observed during the field work that a substantial number of health workers were late to work. However, as per our operational definition, the study did not consider coming late to work as absenteeism. Also, we did not differentiate between the contractual and regular staff in our study. Capturing these differences could have led to further insights. The qualitative phase explored largely the dimensions contributing to health worker absenteeism; we did not explore the dimensions contributing to health worker shortage.

## Conclusion

This study’s results reflect the health worker absenteeism as not merely an individual attribute but a manifested response to systemic challenges and unmet personal and professional needs. While evidence suggests that using stringent human resource management strategies can reduce absenteeism, it may be noted that the systematic issues should not be left unaddressed to sustainably address health worker absenteeism. In Jashpur District of Chhattisgarh, with close to 30% of vacant positions across the healthcare facilities and health worker cadres, the chronic shortage of the health workers can result in overburdening and burnout among existing health workers. Strengthening the health system through infrastructure development, recruiting and retaining health workers, and building necessary support systems are essential to improve efficiency in health system and reduce health worker absenteeism.

## Supporting information

Kerketta et al. supplementary material Kerketta et al. supplementary material

## Supplementary material

The supplementary material for this article can be found at https://doi.org/10.1017/S1463423624000343.
