# Supplementary material for: Exploring health worker absenteeism at public healthcare facilities in Chhattisgarh, India
Source: Prim Health Care Res Dev. 2024 Oct 17;25:e44. doi: 10.1017/S1463423624000343 (PMC11569847; doi:10.1017/S1463423624000343)
Supplement: Kerketta et al. supplementary material [file S1463423624000343sup001.docx]

Supplementary file 1

| **Primary Health Centers (PHC)** | | | |
| --- | --- | --- | --- |
| **Cadre** | **Essential capacity per PHC** | **Total across PHCs** | **Total Positions filled** |
| Medical Officer (MBBS) | 1 | 34 | 8 |
| Medical officer (AYUSH) | 1* | 34 | 26 |
| Rural Medical Assistant | 1 | 34 | 34 |
| Accountant cum Data entry operator | 1 | 34 | 13 |
| Pharmacist | 1 | 34 | 31 |
| Pharmacist AYUSH | 1* | 34 | 7 |
| Staff Nurse | 3 | 102 | 86 |
| Health Worker (Female) | 1 | 34 | 30 |
| Health Assistant (Male) | 1 | 34 | 21 |
| Lady Health Visitor | 1 | 34 | 15 |
| Health Educator | 1* | 34 | 0 |
| Laboratory Technician | 1 | 34 | 26 |
| Cold Chain and Vaccine Logistic Assistant | 1* | 34 | 0 |
| Multi Skilled Group D worker | 2 | 68 | 19 |
| Sanitary worker cum watchman | 1 | 34 | 23 |
| Total | | 612 | 339 |

*Desired

| **Community Health Centers (CHC) (n=8)** | | | |
| --- | --- | --- | --- |
| **Cadre** | **Essential Capacity per CHC** | **Total capacity across CHC** | **Filled** |
| Block Medical Officer | 1 | 8 | 8 |
| Public Health Specialist | 1 | 8 | 0 |
| Public Health Nurse | 1 | 8 | 0 |
| General Surgeon | 1 | 8 | 5 |
| Physician | 1 | 8 | 8 |
| Obstetircian & Gynecologist | 1 | 8 | 8 |
| Pediatrician | 1 | 8 | 2 |
| Anestetist | 1 | 8 | 0 |
| Dental Surgeon | 1 | 8 | 4 |
| General duty Medical officer | 2 | 16 | 16 |
| Medical Officer AYUSH | 1 | 8 | 8 |
| Staff Nurse | 10 | 80 | **85** |
| Pharmacist | 1 | 8 | 8 |
| Pharmacist AYUSH | 1 | 8 | 8 |
| Lab technician | 2 to 3 | 18 | **18** |
| Radiographer | 1 | 8 | 7 |
| Dietician | 1* | 8 | 0 |
| Ophthalmic Assistant | 1 | 8 | 0 |
| Dental Assistant | 1 | 8 | 0 |
| Cold Chain and Vaccine Logistic Assistant | 1 | 8 | 8 |
| OT Technician | 1 | 8 | 7 |
| Multi Rehabilitation/Community Based Rehabilitation worker | 1 | 8 | 0 |
| Counsellor | 1 | 8 | 0 |
| Registration Clerk | 2 | 16 | 14 |
| Statistic Assistant/Data entry operator | 2 | 16 | 16 |
| Account Assistant | 1 | 8 | 8 |
| Adminstrative Assistant | 1 | 8 | 0 |
| Dresser | 1 | 8 | 8 |
| Ward Boy | 5 | 40 | 28 |
| Driver | 1 to 3 | 11 | 11 |
| Total | | 381 | 285 |

*Desired

| **District Hospital (100 Bedded) (n=1)** | | | |
| --- | --- | --- | --- |
| **Cadre** | **Essential Capacity** | **Total Capacity** | **Capacity filled** |
| Medical officer | 11 | 11 | 11 |
| Specialist-Medicine | 2 | 2 | 2 |
| Specialist-Surgery | 2 | 2 | 2 |
| Specialist-Obstetrics & Gynecology | 2 | 2 | 1 |
| Specialist-Pediatrics | 2 | 2 | 2 |
| Specialist-Anesthesia | 2 | 2 | 2 |
| Specialist-Opthalmology | 1 | 1 | 1 |
| Specialist-Orthopedics | 1 | 1 | 1 |
| Specialist-Radiology | 1 | 1 | 1 |
| Specialist-Pathology | 1 | 1 | 1 |
| Specialist-ENT | 1 | 1 | 1 |
| Dental | 1 | 1 | 1 |
| Specialist-Psychiatry | 1* | 1 | 0 |
| Specialist-Dematology | 1* | 1 | 0 |
| Specialist-Microbiology | 1* | 1 | 0 |
| Forensic Specialist | 1* | 1 | 0 |
| AYUSH Doctor | 1 | 1 | 1 |
| Staff Nurse | 45 | 45 | 36 |
| Lab Technician | 6 | 6 | 6 |
| Pharmacist | 5 | 5 | 5 |
| Store Keeper | 1 | 1 | 1 |
| Radiographer | 2 | 2 | 1 |
| ECG/ECHO technician | 1 | 1 | 1 |
| Opthalmic Assistant | 1 | 1 | 0 |
| Dietcian | 1 | 1 | 1 |
| Physiotherapist | 1 | 1 | 1 |
| OT technician | 1 | 1 | 1 |
| CSSD Assistant | 1 | 1 | 1 |
| Social Worker | 2 | 2 | 0 |
| Counsellor | 1 | 1 | 1 |
| Dental Technician | 1 | 1 | 1 |
| Darkroom Assistant | 2 | 2 | 0 |
| Rehabilitation Therapist | 1 | 1 | 0 |
| Biomedical Engineer | 1 | 1 | 0 |
|  | 105 | 105 | 83 |
